# Body composition-derived principal components partially explain sex and age effects on bone mineral density in type 2 diabetes mellitus

**DOI:** 10.3389/fendo.2026.1811776

**Published:** 2026-06-03

**Authors:** Dihe Cheng, Yan Chen, Yan Cai, Jiaxin Wang, Shuangzhu Yang, Junwen Mao, Yanjun Wang

**Affiliations:** Department of Endocrinology, The Second Hospital of Jilin University, Changchun, Jilin, China

**Keywords:** body composition, bone mineral density, mediation analysis, principal component analysis, type 2 diabetes mellitus

## Abstract

**Objective:**

Patients with type 2 diabetes (T2DM) often exhibit high fracture risk. While body composition critically influences bone mineral density (BMD), the specific independent roles of its interrelated components (muscle vs. fat) remain poorly characterized in T2DM. This study aimed to disentangle these correlated phenotypes and quantify their distinct associations with BMD.

**Methods:**

In this cross-sectional study at the Second Hospital of Jilin University, 424 adults with T2DM were assessed. BMD was measured by dual-energy X-ray absorptiometry (DXA), and body composition by bioelectrical impedance analysis (BIA). PCA was applied to derive integrated phenotypes. Multivariable linear regression and mediation analysis assessed their associations with site-specific BMD and quantified the extent to which they statistically account for age/sex effects. A nomogram was developed and internally validated.

**Results:**

The cohort (mean age 58.09 years, 51.4% male, 16.0% osteoporosis prevalence) exhibited significant sex-related differences. PCA mainly identified a “muscle-metabolic” component (PC1) and a “fat-obesity” component (PC2). PC1 was consistently associated with higher BMD at all sites, while PC2 showed a positive association only with total hip BMD, and this association was observed specifically in middle-aged women. PC1 statistically accounted for 30.2–54.5% of the sex effect and 16.7–22.4% of the age effect on BMD. The nomogram (incorporating PC1, PC2, age, sex, and smoking status) demonstrated excellent discrimination (bootstrap-corrected C-index=0.854).

**Conclusions:**

Integrated body composition phenotypes are strongly associated with BMD and statistically explain key demographic effects in T2DM. The developed model provides a cross-sectional tool for identifying current osteoporosis status.

## 1 Introduction

The global burden of type 2 diabetes mellitus (T2DM) continues to rise substantially, with its prevalence projected to reach 7, 079 per 100, 000 individuals by 2030 (1). Impaired bone health, a major complication of T2DM, manifests as a markedly increased risk of fracture—a finding supported by meta-analytic evidence indicating a positive association with overall fracture risk (summary relative risk = 1.05) (2). This contributes to reduced quality of life and heightened healthcare demand in affected individuals. Although a well-recognized paradox in T2DM is the elevated fracture risk despite normal or high bone mineral density (BMD) (3), BMD remains the most widely used and fundamental tool for assessing skeletal health and diagnosing osteoporosis (4). Understanding the determinants of BMD in T2DM is therefore crucial for early identification and management of osteoporosis risk.

Body composition is considered a key determinant of BMD in T2DM (5–7); however, the complex interplay of its components complicates our understanding of how muscle and fat distinctly affect bone density in these patients. Previous studies have primarily examined isolated body composition metrics (e.g., body fat mass, muscle mass, and visceral fat area [VFA]) in relation to BMD (8–10). The combined physiological effects of muscle-metabolic and fat-obesity components on BMD remain unclear, particularly across sex and age groups. Therefore, this study was designed to clarify these relationships through principal component analysis (PCA), which extracts holistic body composition patterns from multiple correlated metrics. This approach allows us to explain the distinct and combined physiological correlations through which muscle and fat influence BMD, contributing to a more comprehensive understanding of skeletal health in T2DM. Building on these insights, we further constructed and validated a nomogram for cross-sectional identification of osteoporosis status in the T2DM population.

## 2 Materials and methods

### 2.1 Study design and participants

This was a hospital-based, cross-sectional study conducted at the Endocrinology Ward of The Second Hospital of Jilin University, a tertiary care center. Participant enrollment and data collection took place between May 11, 2024, and May 11, 2025. All patients diagnosed with T2DM who visited the ward for comprehensive complication screening during the study period were initially considered. The inclusion required the availability of complete BMD and body composition data. Exclusion criteria were age under 18 years at the time of BMD measurement, chronic renal failure or renal dysfunction (estimated glomerular filtration rate [eGFR] < 30 ml/min), chronic liver disease or liver dysfunction (alanine aminotransferase [ALT] > 120 U/L), history of thyroid disease or thyroid surgery, history of cancer, chronic infection, heart failure, or malnutrition, current or previous use of anti-osteoporosis drugs, and current or previous glucocorticoid or sex hormone therapy. A total of 461 patients were initially screened. Following the application of inclusion and exclusion criteria, 424 participants (218 males, 206 females) formed the final analytical cohort (see **Supplementary Figure 1** for the flow diagram). As an exploratory analysis aiming to derive patterns and associations, a formal sample size calculation was not performed *a priori*. Instead, we utilized a consecutive sampling approach to enroll all eligible patients during the study period to maximize the sample size and representativeness within our clinical setting. The study was approved by the Ethics Committee of the Second Hospital of Jilin University (2025 Yan Shen No. 304). Due to the retrospective and anonymous nature of the data, the committee waived the requirement for individual informed consent.

### 2.2 Data collection

Participants underwent a standardized assessment protocol on the same day. After an overnight fast, participants first completed the questionnaire, followed by BMD and body composition measurements. Fasting blood and urine samples were then collected.

Demographic and clinical data (age, sex, smoking history, past medical history) were collected via standardized questionnaires. Smoking status was classified as ‘smoking’ (former/current) or ‘never smoking’. Diabetes duration was calculated from the initial diagnosis.

Glycated hemoglobin A1c (HbA1c) was measured using a fully automated hemoglobin analyzer (HLC-723G8; Tosoh Corporation, Japan). Biochemical analyses were performed using an automated biochemistry analyzer (UniCel DxC 800 Synchron; Beckman Coulter, USA), including liver function tests [e.g., ALT and aspartate aminotransferase (AST)], renal function tests [e.g., creatinine (Cr) and blood urea nitrogen (BUN)], fasting plasma glucose (FPG), lipid profiles [triglycerides (TG), total cholesterol (TC), high-density lipoprotein cholesterol (HDL-C), and low-density lipoprotein cholesterol (LDL-C)], uric acid (UA), and electrolytes (sodium, potassium, calcium, chloride, phosphorus, magnesium). The urinary albumin-to-creatinine ratio (UACR) was measured using a protein analyzer (IMMAGE 800; Beckman Coulter, USA). Thyroid function tests [triiodothyronine (T3), thyroxine (T4), and thyroid-stimulating hormone (TSH)], C-peptide (fasting, 1-hour, and 2-hour), and 25-hydroxyvitamin D were determined using an electrochemiluminescence immunoassay analyzer (cobas e 602; Roche Diagnostics, Switzerland).

BMD was measured at the L1–L4 lumbar spine, femoral neck, and total hip using dual-energy X-ray absorptiometry (DXA; GE Healthcare, USA). All BMD measurements were performed by trained and certified physicians from the Department of Nuclear Medicine. Body composition parameters, including inorganic salts (kg), skeletal muscle mass (kg), skeletal muscle index (SMI, kg/m²), basal metabolic rate (BMR, kcal/day), VFA (cm²), body mass index (BMI, kg/m²), body fat percentage (%), total body water (L), and protein mass (kg), were assessed using a bioelectrical impedance analysis (BIA) device (BIA-120, Chioy Corporation, China). Measurements were performed according to the manufacturer’s standard protocol.

### 2.3 Data analysis

Statistical analyses were performed using SPSS (version 31.0, IBM Corp., USA) and R (version 4.4.0, R Foundation for Statistical Computing, Austria). Continuous variables are presented as mean ± standard deviation or median (interquartile range). Categorical variables are presented as frequencies (percentages). The Student t-test or the Mann–Whitney U test was used for those comparisons of continuous variables, and the chi-square test was used for categorical variables. Missing baseline laboratory data were handled by multiple imputation with chained equations (MICE). Most variables had a missing rate below 10%. Five imputed datasets were created, using predictive mean matching for continuous variables and logistic regression for binary variables. All core analysis variables were fully completed. Statistical significance was defined as two-sided P < 0.05.

PCA included a combination of absolute measures (skeletal muscle mass, protein mass, inorganic salts, BMR, VFA, body fat percentage, and total body water) and standardized indices (BMI, SMI). This hybrid approach was intentional, allowing simultaneous characterization of body size (relevant to mechanical loading) and relative body composition (relevant to metabolic status). All nine body composition variables were standardized to mean = 0 and standard deviation = 1 prior to analysis, and PCA was conducted on the correlation matrix to eliminate dimensional differences and avoid overweighting high-variance indicators. Principal components with eigenvalues >1, confirmed by a scree plot, were retained and subjected to varimax rotation. Individual component scores were calculated via regression scoring using statistical software, and the corresponding component score coefficient matrix is presented in **Supplementary Table 1**.

Multivariable linear regression models were constructed for each skeletal site (lumbar spine, femoral neck, total hip). The retained PCs were entered as independent variables. Diabetes duration was forced into the model based on clinical relevance, and additional covariates (age, sex, smoking status) were included (p < 0.05). Variance inflation factors (VIF) indicated no significant multicollinearity (VIF < 3.0). The potential nonlinear relationship between PCs and BMD was explored using restricted cubic splines (RCS).

Mediation analyses were performed to assess indirect effects of sex and age on site-specific BMD using PCs. The indirect effect (mediation effect) and its 95% confidence interval were estimated, and the proportion statistically accounted for was calculated.

Osteoporosis was defined as the outcome according to the criteria: T-score ≤ -2.5 for postmenopausal women or men ≥50 years, and Z-score ≤ -2.0 for premenopausal women or men <50 years. As an exploratory analysis, a logistic regression model incorporating age, sex, smoking status, PC1, and PC2 was developed and presented as a nomogram for cross-sectional classification of osteoporosis status. Its performance was evaluated by the bootstrap-corrected concordance index (C-index), calibration curves, and decision curve analysis (DCA).

## 3 Results

### 3.1 Participant characteristics

**Table 1** summarizes the baseline characteristics of the total study population (N = 424), which had a mean age of 58.09 ± 10.64 years and a mean diabetes duration of 8.81 ± 7.73 years, comprising 218 (51.4%) males and 206 (48.6%) females. The overall prevalence of osteoporosis was 16.0% (68/424). Of these 68 cases, 67 were diagnosed by T-score criteria and 1 by Z-score criteria. Males were younger (56.65 ± 10.71 vs. 59.62 ± 10.36 years, p = 0.004) and more likely to smoke (52.8% vs. 4.4%, p < 0.001). Marked sex-related differences in body composition were observed: males had higher lean mass measures, including inorganic salts (3.94 ± 0.45 vs. 2.96 ± 0.34 kg), skeletal muscle mass (54.66 ± 6.61 vs. 40.45 ± 4.65 kg), SMI (8.1 ± 0.76 vs. 6.96 ± 0.64 kg/gm²), protein mass (11.33 ± 1.42 vs. 9.22 ± 7.73 kg), and BMR (1595.26 ± 264.22 vs. 1290.16 ± 139.28 kcal; all p < 0.001). Females had higher body fat percentage (33.29 ± 6.48 vs. 25.76 ± 6.55%, p < 0.001) and VFA (111.71 ± 41.32 vs. 101.57 ± 42.01 cm², p = 0.013) and lower BMI (25.48 ± 3.33 vs. 26.48 ± 3.54 kg/m², p = 0.003). The prevalence of osteoporosis was significantly lower in males (4.6% vs. 28.2%). BMD was also significantly higher in males at all sites: L1–L4 lumbar spine (1.21 ± 0.18 vs. 1.06 ± 0.19 g/cm²), femoral neck (0.93 ± 0.15 vs. 0.83 ± 0.14 g/cm²), and total hip (1.01 ± 0.15 vs. 0.90 ± 0.15 g/cm²; all p < 0.001). Males also had higher 25-hydroxyvitamin D levels (49.69 ± 21.32 vs. 45.22 ± 19.99 nmol/L, p = 0.027), while calcium, eGFR, and diabetes duration were similar between groups.

**Table 1:** Baseline characteristics of patients with type 2 diabetes at The Second Hospital of Jilin University.

Characteristics	ALL (n=424)	Males (n=218)	Females (n=206)	P-value
Age (years)	58.09 ± 10.64	56.65 ± 10.71	59.62 ± 10.36	0.004^**^
Smoking, n (%)				<0.001^***^
Never	300 (70.8)	103 (47.2)	197 (95.6)	
former/current	124 (29.2)	115 (52.8)	9 (4.4)	
Diabetes duration (years)	8.81 ± 7.73	9.13 ± 7.47	8.47 ± 8.01	0.381
25-hydroxyvitamin D (nmol/L)	47.52 ± 20.78	49.69 ± 21.32	45.22 ± 19.99	0.027^*^
Calcium (mmol/L)	2.29 ± 0.1	2.29 ± 0.09	2.29 ± 0.1	0.690
eGFR (ml/min)	94.44 ± 16.17	93.77 ± 16.38	95.15 ± 15.95	0.378
Body composition				
Inorganic salts (kg)	3.46 ± 0.63	3.94 ± 0.45	2.96 ± 0.34	<0.001^***^
Skeletal muscle mass (kg)	47.75 ± 9.13	54.66 ± 6.61	40.45 ± 4.65	<0.001^***^
SMI (kg/gm²)	7.54 ± 0.91	8.1 ± 0.76	6.96 ± 0.64	<0.001^***^
Protein mass (kg)	10.30 ± 5.58	11.33 ± 1.42	9.22 ± 7.73	<0.001^***^
BMR (kcal/day)	1447.03 ± 261.77	1595.26 ± 264.22	1290.16 ± 139.28	<0.001^***^
Body fat percentage (%)	29.42 ± 7.52	25.76 ± 6.55	33.29 ± 6.48	<0.001^***^
VFA (cm²)	106.49 ± 41.94	101.57 ± 42.01	111.71 ± 41.32	0.013^*^
BMI (kg/m^2^)	25.99 ± 3.47	26.48 ± 3.54	25.48 ± 3.33	0.003^**^
Total body water (L)	39.34 ± 24.24	44.33 ± 23.34	34.06 ± 24.10	<0.001^***^
Bone mineral density				
L1-L4 Lumbar(g/cm^2^)	1.14 ± 0.2	1.21 ± 0.18	1.06 ± 0.19	<0.001^***^
Femoral Neck(g/cm^2^)	0.88 ± 0.15	0.93 ± 0.15	0.83 ± 0.14	<0.001^***^
Total Hip(g/cm^2^)	0.96 ± 0.16	1.01 ± 0.15	0.9 ± 0.15	<0.001^***^
Osteoporosis risk, n (%)	68 (16.0)	10 (4.6)	58 (28.2)	<0.001^***^

^a^ Data are presented as mean ± standard deviation or number (percentage). Between-group comparisons were made using an independent samples t-test for continuous variables and a χ² test for categorical variables.

^b^ Significance: ^***^p<0.001, ^**^p<0.01, ^*^p<0.05.

^c^ SMI, skeletal muscle index; BMR, basal metabolic rate; VFA, visceral fat area; BMI, body mass index; eGFR, estimated glomerular filtration rate.

### 3.2 PCA of body composition

PCA applied to this comprehensive set of variables successfully distilled complex data into interpretable components, collectively explaining 89.22% of total variance, supported by the scree plot (**Supplementary Figure 2**). Component loadings (**Supplementary Table 2**) provided clear physiological interpretations: PC1 (43.59% of variance), characterized by high loadings for inorganic salts, skeletal muscle mass, SMI, and BMR, was termed the “muscle-metabolic” component. PC2 (27.61% of variance), loaded strongly on VFA, body fat percentage, and BMI, was termed the “fat-obesity” component. PC3 (18.02% of variance) was primarily driven by total body water and protein mass, representing a “Fluid-Protein” component.

**Figure 1:**
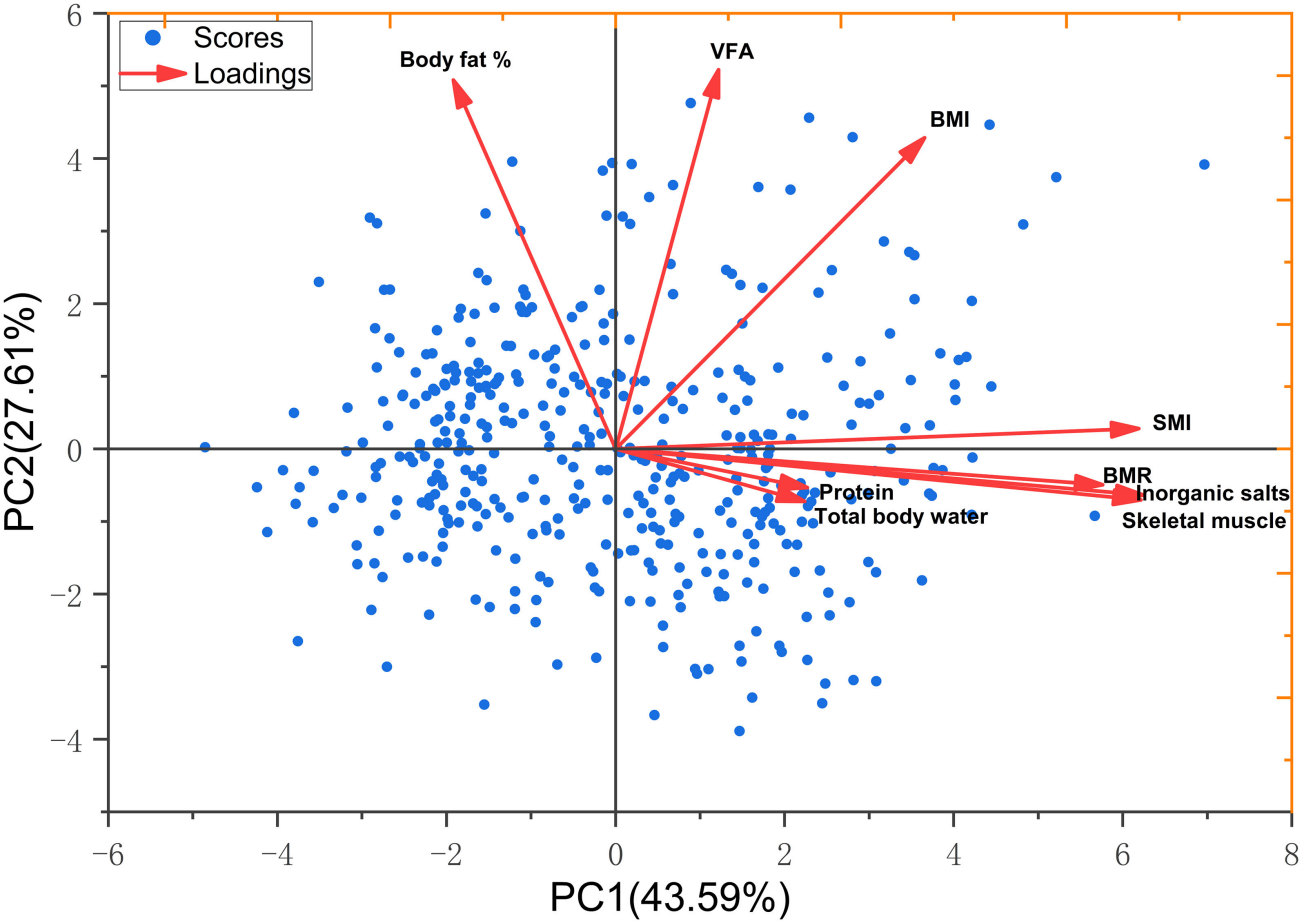
PCA biplot of body composition features. The biplot displays the joint distribution of component scores (scatter points) and variable loadings (vectors) for the first two principal components (PC1 and PC2) derived from the principal component analysis. PC1 and PC2 explain 43.59% and 27.61% of the total variance, respectively. The direction of the vectors indicates the correlation strength between variables and components, while their length represents the contribution magnitude. The distribution of sample points reflects inter-individual differences in the PC1(muscle-metabolic) and PC2(fat-obesity) body composition patterns; PCA, principal component analysis; PC, principal component; VFA, visceral fat area; BMI, body mass index; SMI, skeletal muscle index; BMR, basal metabolic rate.

Given their clear pathophysiological relevance and substantial cumulative variance (71.20%), PC1 and PC2 were retained for subsequent analyses. The PCA biplot (**Figure 1**) showed a distinct separation of variables, with muscle-metabolic parameters clustering along the positive PC1 axis and fat-obesity parameters aligning along the positive PC2 axis.

### 3.3 Associations between body composition PCs and BMD

Multivariate linear regression adjusted for age, sex, smoking status, and diabetes duration revealed independent associations between the principal body composition components (PC1 and PC2) and BMD (**Table 2**). PC1 was a significant positive predictor of BMD at all sites–L1–L4 lumbar (β= 0.180, p < 0.05), femoral neck (β = 0.260, p < 0.001), and total hip (β = 0.288, p < 0.001). PC2 was positively associated only with total hip BMD (β = 0.103, p < 0.05). RCS analysis confirmed the linearity of these associations (p for non-linearity > 0.05; **Supplementary Figure 3**). Associations between adjusted covariates (age, sex, smoking status, and diabetes duration) and BMD are presented in **Supplementary Table 3**.

**Table 2:** Associations of body composition principal components with bone mineral density in this study: total population and subgroup analyses.

Total/Subgroup		PC1 β(P)			PC2 β(P)		
	N	L1-L4 Lumbar BMD	Femoral Neck BMD	Total Hip BMD	L1-L4 Lumbar BMD	Femoral Neck BMD	Total Hip BMD
Total	424	0.180 (0.012) ^*^	0.260 (<0.001)^***^	0.288 (<0.001) ^***^	0.089 (0.059)	0.062 (0.163)	0.103 (0.023) ^*^
Sex Group							
Male	218	0.163 (0.033) ^*^	0.240 (0.001) ^**^	0.263 (0.000) ^***^	0.101 (0.159)	0.010 (0.890)	0.082 (0.237)
Female	206	0.143 (0.038) ^*^	0.150 (0.022) ^*^	0.180 (0.006) ^**^	0.100 (0.131)	0.140 (0.026) ^*^	0.150 (0.018) ^*^
Age Group							
<50 years	83	0.281 (0.169)	0.479 (0.015) ^*^	0.328 (0.101)	-0.096 (0.467)	0.040 (0.746)	0.024 (0.851)
50~<65 years	221	0.230 (0.020) ^*^	0.287 (0.004) ^**^	0.303 (0.002) ^**^	0.175 (0.010) ^*^	0.093 (0.173)	0.152 (0.024) ^*^
≥65 years	120	0.165 (0.092)	0.245 (0.025) ^*^	0.334 (0.002) ^**^	0.085 (0.252)	0.026 (0.757)	0.112 (0.159)

^a^ All models are adjusted for age, sex, smoking, and diabetes duration, except for the sex-stratified models (adjusted for age, smoking, duration) and age-stratified models (adjusted for sex, smoking, duration).

^b^ β, standardized beta coefficient.

^c^ Significance: ^***^p<0.001, ^**^p <0.01, ^*^p <0.05.

Sex-stratified analyses revealed notable differences (**Table 2**). Stratified heat maps (**Supplementary Figure 4**) provided detailed visualizations. PC1 showed consistent positive associations across all skeletal sites in both sexes, with a tendency toward stronger effects in men. In contrast, PC2 demonstrated significant positive associations with femoral neck and total hip BMD in women, but not in men.

Age-stratified analyses identified distinct trends (**Table 2**; **Supplementary Figure 4**). With advancing age, the association between PC1 and BMD gradually weakened in the L1–L4 lumbar spine and femoral neck but strengthened at the total hip. For PC2, significant positive associations with lumbar and total hip BMD were observed only in participants aged 50 to<65 years, with no significant associations in other age groups.

### 3.4 Statistical explanatory role of body composition in age/sex effects on BMD

**Figure 2:**
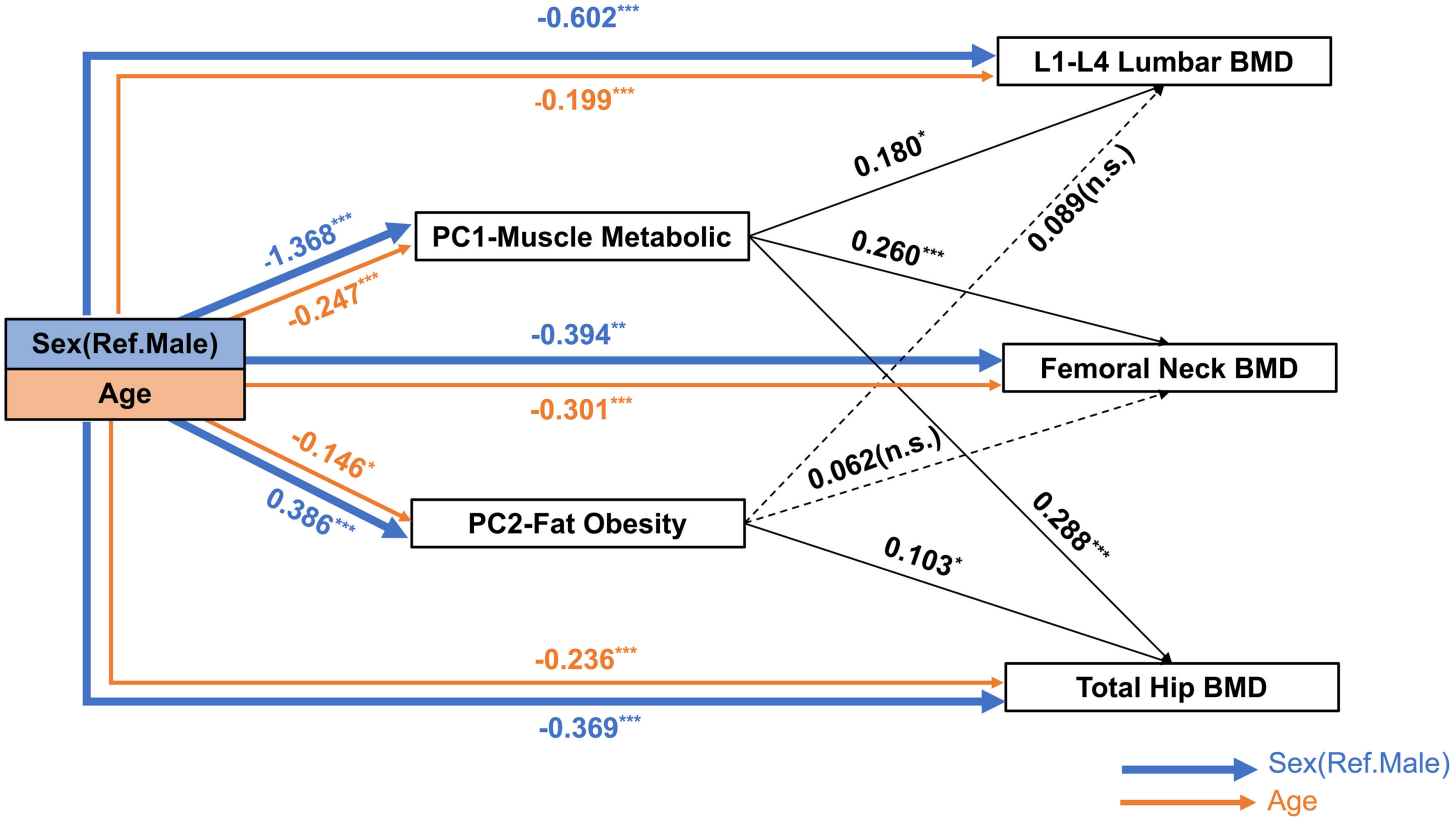
Statistical explanatory role of body composition in age/sex-BMD relationships. The model shows that sex and age are significantly associated with both body composition components, but only PC1 consistently accounts for a portion of their statistical effects on all BMD sites; Path coefficients are standardized with significance levels denoted by asterisks (^*^p< 0.05, ^**^p < 0.01, ^***^p < 0.001) and n.s. (not significant, confidence interval includes 0).

Mediation analysis suggested that body composition statistically accounts for a substantial portion of both sex and age effects on BMD (**Figure 2**). Compared with males, female sex had significant total negative effects on BMD at all sites (p < 0.001; **Supplementary Table 4**) and was strongly associated with lower PC1 (β = -2.681, p < 0.001) and higher PC2 (β= 0.614, p = 0.001; **Supplementary Table 5**). Effect decomposition revealed significant indirect effects through PC1 at all sites, accounting for 30.2% (L1–L4 Lumbar), 49.0% (Femoral Neck), and 54.5% (Total Hip) of the total effects (**Supplementary Table 4**). PC2 showed a limited statistical explanatory role, contributing significantly only to total hip BMD (indirect effect = 0.0064; 95% CI: 0.001–0.014) (**Supplementary Table 4**).

Older age also had significant total negative effects on BMD (all *p* < 0.001; **Supplementary Table 6**) and was associated with lower PC1 (*β* = -0.046, *p* < 0.001) and PC2 (*β* = -0.022, *p* = 0.006; **Supplementary Table 5**). Significant indirect effects through PC1 were observed at all skeletal sites, statistically accounting for 16.7–22.4% of the total effects (**Supplementary Table 6**). A small but significant indirect effect via PC2 was also found only for total hip BMD.

### 3.5 Development and validation of a cross−sectional diagnostic model

**Figure 3:**
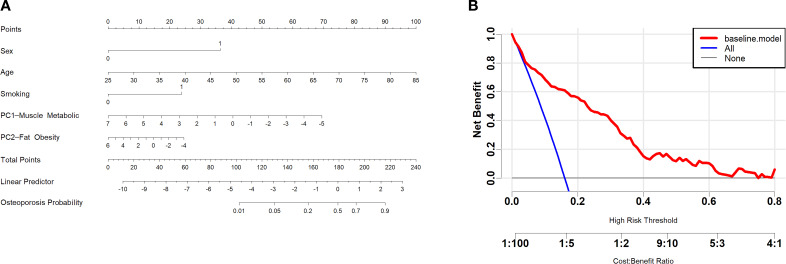
Nomogram and decision curve analysis for cross−sectional identification of osteoporosis, **(A)** Nomogram for estimating osteoporosis probability. Locate patient values on each variable axis to determine points, sum total points, and project to the probability axis; Variable coding: Sex (1, Female; 0, Male); Smoking (1, Former or current smoker; 0, Never smoker); **(B)** Decision curve analysis shows the net benefit of using the nomogram for screening decisions compared to ‘ Diagnose All ‘ and ‘ Diagnose None ‘ strategies across threshold probabilities of 1–50%.

**Figure 3A** shows the nomogram. The total score is calculated by summing the corresponding score points. The model demonstrated good discrimination, with a bootstrap-corrected C-index of 0.854, indicating minimal overfitting (optimism = 0.016). Its potential utility was further supported by DCA (**Figure 3B**), which showed higher net benefit than “diagnose all” or “diagnose none” strategies across threshold probabilities of approximately 1–50%. The model calibration was excellent, with a mean absolute error of 0.019 (**Supplementary Figure 5**), supporting its accuracy despite a significant Hosmer–Lemeshow result (p = 0.037). A sensitivity analysis restricted to T−score−defined participants (n=343) confirmed the robustness of the findings, yielding a bootstrap-corrected C-index of 0.832.

## 4 Discussion

This study aimed to elucidate the complex relationship between multidimensional body composition and BMD in patients with T2DM by applying PCA and to develop a cross-sectional tool for identifying current osteoporosis status. Our key findings are as follows: First, PCA successfully mainly distilled highly correlated body composition measures into two physiologically distinct and interpretable components: a “muscle-metabolic” component (PC1) and a “fat-obesity” component (PC2). Second, these components showed differential associations with BMD: PC1 was consistently positively associated with BMD across all skeletal sites, whereas the association of PC2 was positive, site-specific, with observations differing across sex and age groups. Third, mediation analysis revealed that these body composition patterns significantly explain a substantial portion of the known effects of both sex and aging on BMD. Finally, integrating these insights into a nomogram provided a well-validated model for cross-sectional identification of osteoporosis status in T2DM.

PCA-derived PC1 (the muscle-metabolic component) was a consistent, positive correlate of BMD across all skeletal sites. This reinforces the established physiological link between muscle and bone, likely mediated by mechanical loading and myokine secretion (11). Our finding extends the observation by Akeroyd et al., which linked lower lean mass in men with T2DM to fracture risk primarily through non-skeletal (e.g., fall-related) pathways (12), by demonstrating that a holistic muscle-metabolic phenotype directly correlates with BMD itself. This association was stronger in men and exhibited a distinct site-specific pattern with aging: while it weakened at the lumbar spine and femoral neck, a trend that is consistent with sarcopenia (13), it strengthened at the total hip, the most common site of osteoporotic fracture (14). This underscores the particular importance of preserving muscle mass for hip bone health in the aging T2DM population. Therefore, our study advances prior evidence by identifying an integrated body composition phenotype that not only corroborates the muscle-bone unit theory but also clarifies its nuanced, site-specific interaction with aging, offering a targeted mechanistic insight for preserving skeletal health in T2DM.

The association of PC2 (the fat-obesity component) with BMD exhibited distinct site-specific patterns, with observations varying across sex and age groups. In the total population, PC2 was positively and significantly associated only with hip BMD. When stratified by sex and age, this association was statistically significant only in women, particularly within the 50-60-year age group.This finding partially aligns with that reported by Shan et al., who observed a positive correlation between BMI and hip BMD in middle-aged female patients with T2DM (15). Our analysis refines this observation by suggesting that it is specifically an obesity-driven fat distribution pattern (captured by PC2), rather than body weight or BMI alone, that is relevant. A possible explanation for the stronger association in middle-aged women involves the mechanical loading effect of greater adiposity on weight−bearing sites such as the hip (16, 17). Additionally, differences between sexes in fat distribution and metabolic responses to chronic hyperglycemia may modify the relationship between fat mass and bone (18, 19). These observations should be interpreted cautiously, as formal interaction tests did not confirm significant effect modification by sex or age. In contrast, the lack of a clear positive association in men and in older adults (≥65 years) suggests that these potential benefits are likely overcome by other detrimental, age- and sex-related processes, including adipose tissue dysfunction and chronic low-grade systemic inflammation (20–22). Therefore, this study moves beyond simple BMI correlations and identifies a specific pattern of fat distribution whose skeletal correlate is most evident in middle−aged women, reflecting heterogeneous interactions between adipose tissue phenotypes and bone metabolism across sex and age in the T2DM population.

A significant novel contribution of this study is the demonstration that the derived body composition components statistically account for a substantial portion of the effects of sex and age on BMD in T2DM. We found that PC1 (the muscle-metabolic component) statistically accounted for approximately 30.2-54.5% of the sex effect and 16.7-22.4% of the age effect on BMD. This finding suggests a potential pathway underlying the established muscle-bone cross-talk (23) and helps explain demographic variations in BMD within the metabolically heterogeneous T2DM population. Specifically, the consistently lower BMD in women and older adults may be partly explained by their tendency toward a less favorable muscle-metabolic phenotype (lower PC1). While previous studies emphasize that poorer physical function or reduced muscle mass, rather than BMD deficits, is a primary driver of fracture risk in T2DM (12, 24), our findings suggest that the muscle-metabolic phenotype may also play a role in both BMD and fracture risk. Extending these mechanistic insights, our study underscores that interventions aimed at preserving or improving the muscle-metabolic phenotype, such as exercise and nutritional support (25–28), may be an effective strategy to maintain bone health in women and older adults with T2DM, potentially reducing risks associated with age and sex.

Building on these observations, we developed an internally validated nomogram incorporating age, sex, smoking status, and the key body composition phenotypes (PC1 and PC2). The model showed strong discrimination (bootstrap-corrected C−index = 0.854). Its exclusion of conventional diabetic factors, such as disease duration, which lacked independent significance, aligns with and may help explain the T2DM BMD paradox (29, 30). By integrating multidimensional body composition derived from routine BIA, our model highlights a modifiable determinant of BMD.

The strengths of this study lie in its integrative approach and translational output. First, by applying PCA, we moved beyond analyzing isolated body composition metrics to defining holistic, physiologically distinct phenotypes that explain a substantial share of demographic disparities in BMD. Second, we assessed the statistical explanatory role of these phenotypes, offering a novel explanatory model for skeletal fragility in T2DM. Finally, the development and validation of a nomogram demonstrate a path for implementing these insights into cross-sectional identification of osteoporosis status in the T2DM population.

Nevertheless, this study had several limitations. First, the cross-sectional design precludes causal inferences, and the focus on Chinese adults with T2DM limits generalizability. Moreover, participants were recruited from an endocrinology ward during complication screening, representing a selected hospital-based sample that may not reflect the broader T2DM outpatient population. Second, the osteoporosis model relies solely on BMD and lacks data on bone quality, such as microarchitecture and collagen cross-links, as well as bone turnover markers such as parathyroid hormone and osteocalcin. Third, the nomogram lacks external validation, and its performance requires confirmation in future studies; furthermore, the PCA components (PC1 and PC2) are derived from the specific rotation matrix of this sample, so the nomogram cannot be directly applied by clinicians without replicating the entire PCA procedure. A simplified scoring algorithm or external validation is needed. Of note, the Hosmer-Lemeshow test yielded a P value of 0.037 (with 68 events and 5 predictors), indicating modest miscalibration, which should be considered when interpreting the model. Fourth, we did not collect information on physical activity level, calcium or vitamin D supplementation, or detailed glucose-lowering medication history (e.g., thiazolidinediones, metformin). These factors are known to influence bone metabolism in T2DM; however, as this was a retrospective study based on routine hospital records, such data were subject to recall bias and lacked standardized documentation in the routine hospital records. Their absence may limit the comprehensiveness of the model. In addition, smoking status was highly confounded with sex in the present cohort, with an obvious difference in smoking prevalence between males and females. Although the variance inflation factor remained within an acceptable range, this close correlation may partially limit the independent interpretability of these two variables in the multivariable model. Additionally, the small number of premenopausal women (n = 29) precluded stratified analyses according to menopausal status, and we adjusted for age across all models to mitigate this potential confounding effect. Therefore, our model is mainly applicable to middle-aged and older adults with T2DM. Future prospective studies are warranted to verify our findings regarding prevalent osteoporosis status and related skeletal outcomes.

In conclusion, this study demonstrates that in patients with T2DM, body composition is best understood as integrated muscle-metabolic and fat-obesity phenotypes, which are differentially associated with BMD in a site-specific manner, with observations differing across sex and age groups. Crucially, these phenotypes statistically account for a significant portion of the effects of sex and aging on bone health. By integrating these phenotypes into a nomogram, we provide a validated tool for cross-sectional identification of osteoporosis in T2DM. Our findings underscore the importance of comprehensive body composition assessment in the management of T2DM and highlight the muscle-metabolic component as a potential key target for strategies aimed at preserving skeletal health in this high-risk population.

## Data Availability

The raw data supporting the conclusions of this article will be made available by the authors, without undue reservation.
