# Supplementary material for: Body composition-derived principal components partially explain sex and age effects on bone mineral density in type 2 diabetes mellitus
Source: Front Endocrinol (Lausanne). 2026 Jun 3;17:1811776. doi: 10.3389/fendo.2026.1811776 (PMC13271991; doi:10.3389/fendo.2026.1811776)
Supplement: Supplementary file 1 [file DataSheet1.pdf]

Supplementary Table S1. Component Score Coefficient Matrix Page 1
Supplementary Table S2: Rotated Component Matrix from Principal Component Analysis of Body Composition Variables Page 2
Supplementary Table S3: Predictors of L1-L4 Lumbar Spine, Femoral Neck, and Total Hip BMD: Non-Standardized Regression Results Page 3
Supplementary Table S4: Decomposition of Total, Direct, and Indirect Effects of Sex on BMD Page 4
Supplementary Table S5: Regression Coefficients of Direct and Statistical Explanatory Models Page 5
Supplementary Table S6: Decomposition of Total, Direct, and Indirect Effects of Age on BMD Page 6
Supplementary Figure S1: Participant Flow Diagram Page 7
Supplementary Figure S2: Scree Plot of the Principal Component Analysis for Body Composition Page 8
Supplementary Figure S3: Nonlinear Associations of Body Composition PC1 and PC2 with BMD Page 9
Supplementary Figure S4: Sex-/ Age-Stratified Heatmap of Body Composition Effects on BMD Page 10
Supplementary Figure S5: Calibration Curve of the Diagnostic Model Page 11

Supplementary Table S1. Component Score Coefficient Matrix

Variable	PC1-Muscle Metabolic	PC2-Fat Obesity	PC3-Fluid Protein
**Inorganic salts (kg)**	0.251	-0.027	-0.026
**Skeletal muscle mass (kg)**	0.250	-0.032	-0.028
**SMI (kg/m²)**	0.241	0.040	-0.030
**BMR (kcal/day)**	0.237	-0.020	-0.047
**VFA (cm²)**	-0.001	0.392	0.001
**BMI (kg/m²)**	0.105	0.329	-0.015
**Body fat percentage (%)**	-0.127	0.372	0.029
**Total body water (L)**	-0.068	0.001	0.574
**Protein mass (kg)**	-0.069	0.016	0.575

[a] Extraction method: Principal Component Analysis. Rotation method: Varimax with Kaiser normalization.

[b] Abbreviations: SMI, Skeletal Muscle Index; BMR, Basal Metabolic Rate; VFA, Visceral Fat Area; BMI, body mass index.

Supplementary Table S2. Rotated Component Matrix from Principal Component Analysis of Body Composition Variables

Variable	PC1-Muscle Metabolic	PC2-Fat Obesity	PC3-Fluid Protein	Communality
**Inorganic salts (kg)**	*0.960*	-0.015	0.128	0.938
**Skeletal muscle mass (kg)**	*0.957*	-0.028	0.126	0.932
**SMI (kg/m²)**	*0.934*	0.150	0.114	0.909
**BMR (kcal/day)**	*0.893*	0.000	0.084	0.804
**VFA (cm²)**	0.078	*0.973*	-0.011	0.953
**BMI (kg/m²)**	0.469	*0.841*	0.037	0.928
**Body fat percentage (%)**	-0.400	*0.898*	-0.050	0.970
**Total body water (L)**	0.121	-0.027	*0.884*	0.798
**Protein mass (kg)**	0.122	0.008	*0.885*	0.798
**Variance explained (%)**	43.59	27.61	18.02	-
**Cumulative %**	43.59	71.20	89.22	-

[a] Loadings with an absolute value > 0.80 are presented in italics for clarity. Extraction Method: Principal Component Analysis. Rotation Method: Varimax with Kaiser Normalization.

[b] Abbreviations: SMI, Skeletal Muscle Index; BMR, Basal Metabolic Rate; VFA, Visceral Fat Area; BMI, body mass index.

Supplementary Table S3. Predictors of L1-L4 Lumbar Spine, Femoral Neck, and Total Hip BMD: Non-Standardized Regression Results

Predictor	L1-L4 Lumbar	Femoral Neck	Total Hip
**Age (years)**	-0.004 (-0.006, -0.002)***	-0.004 (-0.006, -0.003)***	-0.004 (-0.005, -0.002)***
	β=-0.199	β=-0.301	β=-0.236
**Sex (Ref: Male)**	-0.120 (-0.177, -0.063)***	-0.060 (-0.101, -0.018)**	-0.059 (-0.104, -0.015)**
	β=-0.301	β=-0.197	β=-0.185
**Smoking (Ref: Never)**	-0.063 (-0.107, -0.019)**	-0.039 (-0.070, -0.007)*	-0.044 (-0.078, -0.010)*
	β=-0.144	β=-0.116	β=-0.126
**Diabetes Duration (years)**	0.002 (0.000, 0.004)	0.000 (-0.002, 0.002)	0.000 (-0.002, 0.002)
	β=0.081	β=0.007	β=0.007
**PC1-Muscle Metabolic**	0.018 (0.004, 0.032)*	0.020 (0.010, 0.030)***	0.024(0.013, 0.035)***
	β=0.180	β=0.260	β=0.288
**PC2-Fat Obesity**	0.011 (0.000, 0.023)	0.006 (-0.002, 0.014)	0.010 (0.001, 0.019)*
	β=0.089	β=0.062	β=0.103
**Adjusted R^2**	0.222	0.298	0.273

[a] Data presented are unstandardized regression coefficients (B) with 95% confidence intervals (95% CI). Standardized coefficients (β) are shown in the second line. The model included age, sex, smoking, diabetes duration, and body composition principal components (PC1, PC2). PC3 was excluded due to a lack of statistical significance (P >0.05).

[b] The adjusted R^2 represents the proportion of variance in BMD explained by the model after adjusting for the number of predictors. The overall F-test for all models was significant at $P < 0.001$. Variance inflation factors (VIF) were all below 3.0, indicating no evidence of multicollinearity.

[c] Significance: *** P<0.001, ** P<0.01, * P <0.05.

[d] Abbreviations: Ref, reference category; BMD, bone mineral density.

Supplementary Table S4. Decomposition of Total, Direct, and Indirect Effects of Sex on BMD

Outcome	Path	Total Effect	Direct Effect	Indirect Effect	Boot SE	Boot LLCI	Boot ULCI	Std. Indirect Effect	Proportion Mediated
**L1-L4 Lumbar BMD**	Sex → PC1 →L1-L4 Lumbar BMD	-0.1624***	-0.1202***	-0.0490	0.0188	-0.0875	-0.0130	-0.2455	30.2%
	Sex → PC2 →L1-L4 Lumbar BMD	-0.1624***	-0.1202***	0.0068	0.0045	-0.0001	0.0174	0.0342(n.s.)	-4.2%
**Femoral Neck BMD**	Sex → PC1 →Femoral Neck BMD	-0.1100***	-0.0597**	-0.0539	0.0164	-0.0874	-0.0224	-0.3553	49.0%
	Sex → PC2 →Femoral Neck BMD	-0.1100***	-0.0597**	0.0036	0.0029	-0.0014	0.0103	0.0239(n.s.)	-3.2%
**Total Hip BMD**	Sex → PC1 →Total Hip BMD	-0.1162***	-0.0593**	-0.0633	0.0145	-0.0928	-0.0357	-0.3933	54.5%
	Sex → PC2 →Total Hip BMD	-0.1162***	-0.0593**	0.0064	0.0034	0.0010	0.0140	0.0399	-5.5%

[a] Results are presented as unstandardized coefficients with standard errors or bootstrapped estimates. Total and direct effects are reported as β (SE), while the indirect effect is reported as β (BootSE). Effects are considered not significant (n.s.) if the 95% bias-corrected bootstrap confidence interval (based on 5,000 samples) includes zero. The Std. Indirect Effect represents the completely standardized indirect effect (β) for comparison across paths.

[b] Significance: *** P<0.001, ** P<0.01, * P <0.05.

[c] Abbreviations: BMD, bone mineral density; PC, principal component; BootSE, bootstrap standard error; LLCI/ULCI, lower/upper limit of the confidence interval.

Supplementary Table S5. Regression Coefficients of Direct and Statistical Explanatory Models

Variable	L1-L4 Lumbar BMD	Femoral Neck BMD	Total Hip BMD	Mediator 1 (PC1-Muscle Metabolic)	Mediator 2 (PC2-Fat Obesity)
**Sex (Ref: Male)**	-0.120***(0.000)	-0.060**(0.005)	-0.059**(0.009)	-2.681*** (0.000)	0.614**(0.001)
	(-0.177,-0.063)	(-0.101,-0.018)	(-0.104,-0.015)	(-2.969,-2.392)	(0.260,0.969)
**Age**	-0.004*** (0.000)	-0.004***(0.000)	-0.004***(0.000)	-0.046*** (0.000)	-0.022** (0.006)
	(-0.006,-0.002)	(-0.006,-0.003)	(-0.005,-0.002)	(-0.058,-0.033)	(-0.037,-0.006)
**Mediator 1 (PC1-Muscle Metabolic)**	0.018*(0.012)	0.020***(0.000)	0.024***(0.000)	—	—
	(0.004,0.032)	(0.010,0.030)	(0.013,0.035)	—	—
**Mediator 2 (PC2-Fat Obesity)**	0.011(0.059)	0.006(0.163)	0.010*(0.023)	—	—
	(-0.000,0.023)	(-0.002,0.014)	(0.002,0.019)	—	—
**Smoking (Ref: Never)**	-0.063** (0.005)	-0.039*(0.016)	-0.045*(0.011)	0.071(0.656)	0.132(0.503)
	(-0.107,-0.020)	(-0.070,-0.007)	(-0.079,-0.011)	(-0.243,0.385)	(-0.254,0.517)
**Diabetes Duration**	0.002(0.087)	0.000(0.870)	0.000(0.882)	0.006 (0.481)	-0.008(0.435)
	(-0.000,0.005)	(-0.002,0.002)	(-0.002,0.002)	(-0.011,0.023)	(-0.030,0.013)

[a] Regression coefficients are reported as β (standard error). P-values are shown adjacent to coefficients, with 95% confidence intervals provided in brackets below. A dash (—) indicates that the variable was not included in the specific model. All statistical explanatory models were tested using bootstrapping with 5,000 samples to generate bias-corrected confidence intervals for indirect effects.

[b] Significance: *** P<0.001, ** P<0.01, * P <0.05.

[c] Abbreviations: BMD, bone mineral density; PC, principal component; Ref, reference category.

Supplementary Table S6. Decomposition of Total, Direct, and Indirect Effects of Age on BMD

Outcome	Path	Total Effect	Direct Effect	Indirect Effect	Boot SE	Boot LLCI	Boot ULCI	Std. Indirect Effect	Proportion Mediated
**L1-L4 Lumbar BMD**	Increasing age → PC1 →L1-L4 Lumbar BMD	-0.0048***	-0.0037***	-0.0008	0.0003	-0.0016	-0.0002	-0.0444	16.7%
	Increasing age → PC2 →L1-L4 Lumbar BMD	-0.0048***	-0.0037***	-0.0002	0.0002	-0.0006	0.0000	-0.0129 (n.s.)	4.2%
**Femoral Neck BMD**	Increasing age → PC1 →Femoral Neck BMD	-0.0053***	-0.0043***	-0.0009	0.0003	-0.0016	-0.0004	-0.0642	17.2%
	Increasing age → PC2 →Femoral Neck BMD	-0.0053***	-0.0043***	-0.0001	0.0001	-0.0004	0.0001	-0.0091(n.s.)	1.9%
**Total Hip BMD**	Increasing age → PC1 →Total Hip BMD	-0.0049***	-0.0036***	-0.0011	0.0003	-0.0017	-0.0006	-0.0711	22.4%
	Increasing age → PC2 →Total Hip BMD	-0.0049***	-0.0036***	-0.0002	0.0001	-0.0005	0.0000	-0.0151	4.1%

[a] Results are presented as unstandardized coefficients with standard errors or bootstrapped estimates. Total and direct effects are reported as β (SE), while the indirect effect is reported as β (BootSE). Effects are considered not significant (n.s.) if the 95% bias-corrected bootstrap confidence interval (based on 5,000 samples) includes zero. The Std. Indirect Effect represents the completely standardized indirect effect (β) for comparison across paths.

[b] Significance: ***P<0.001, ** P<0.01, * P <0.05.

[c] Abbreviations: BMD, bone mineral density; PC, principal component; BootSE, bootstrap standard error; LLCI/ULCI, lower/upper limit of the confidence interval.

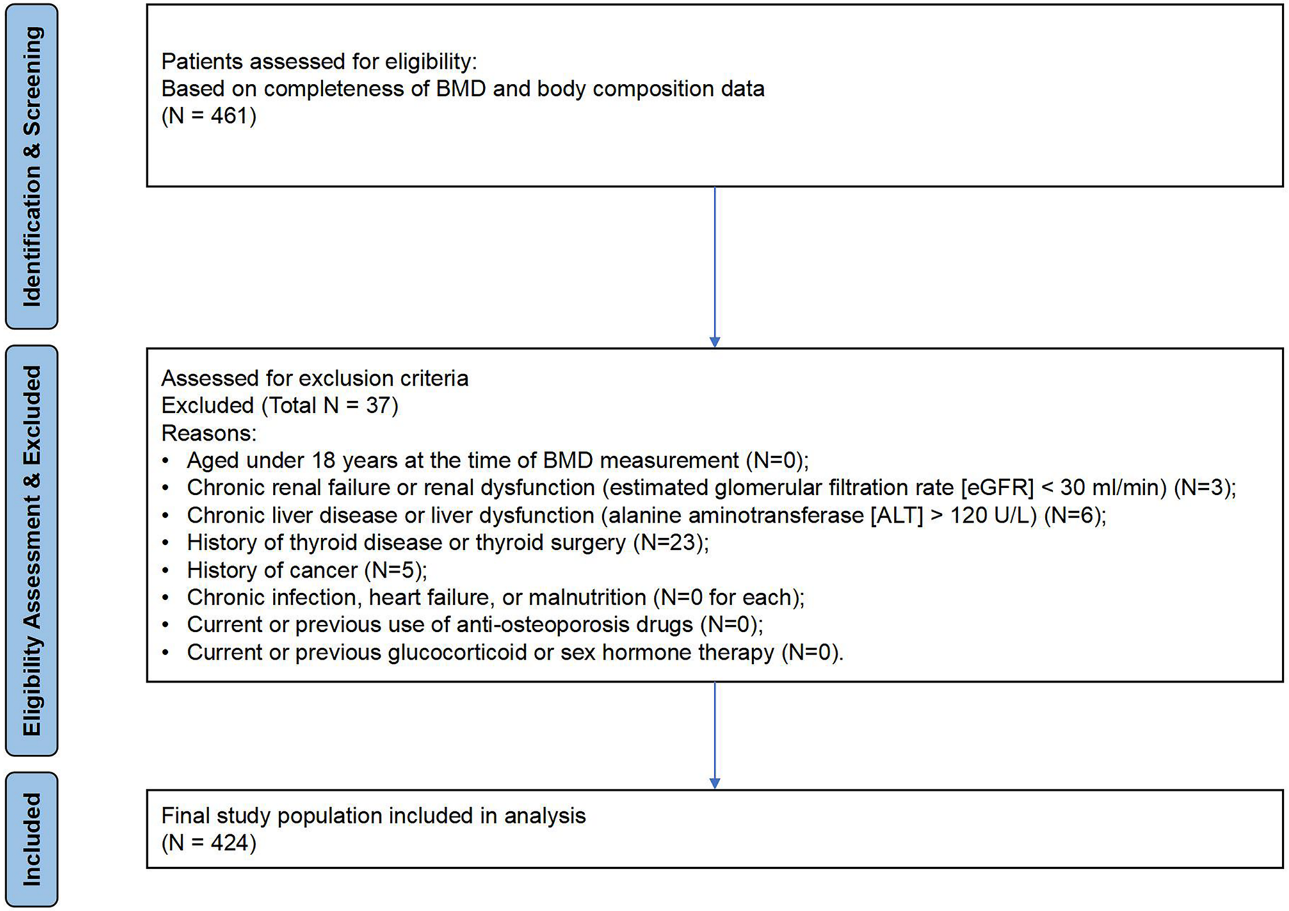

Supplementary Fig. S1 Participant Flow Diagram

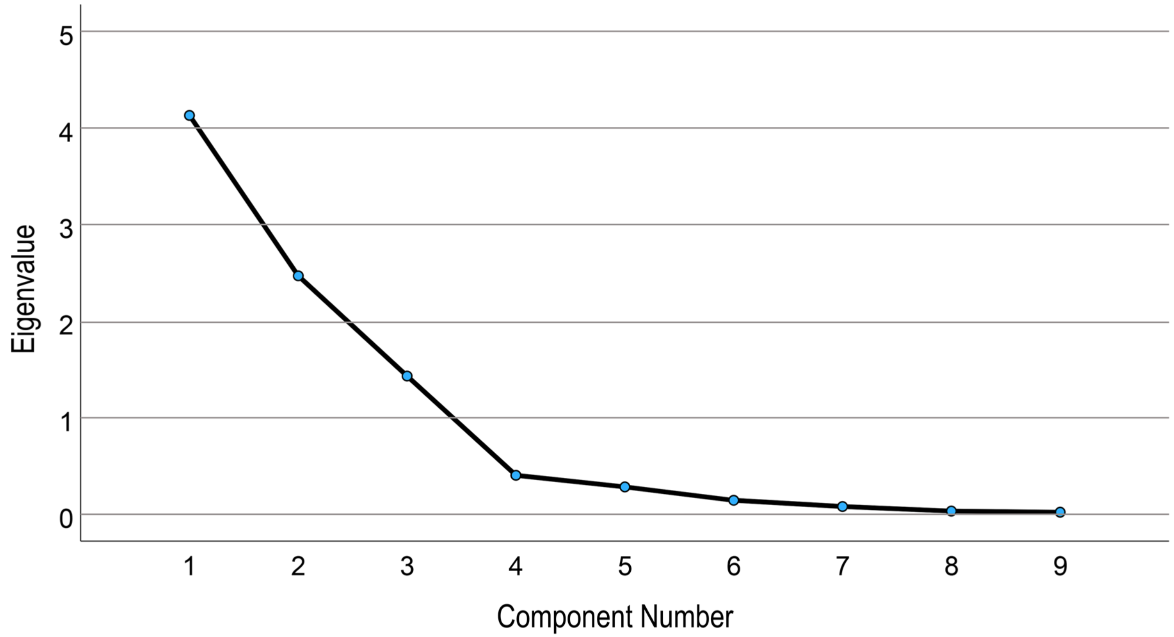

Supplementary Fig. S2 Scree Plot of the Principal Component Analysis for Body Composition
The scree plot displays the relationship between the eigenvalues (y-axis) and the component numbers (x-axis) from the principal component analysis. The plot indicates that the first three components have eigenvalues greater than 1 (Kaiser-Harris criterion reference line), and the curve levels off after the third component (the "elbow" point), supporting the decision to retain three principal components. These three components cumulatively account for 89.22% of the total variance.

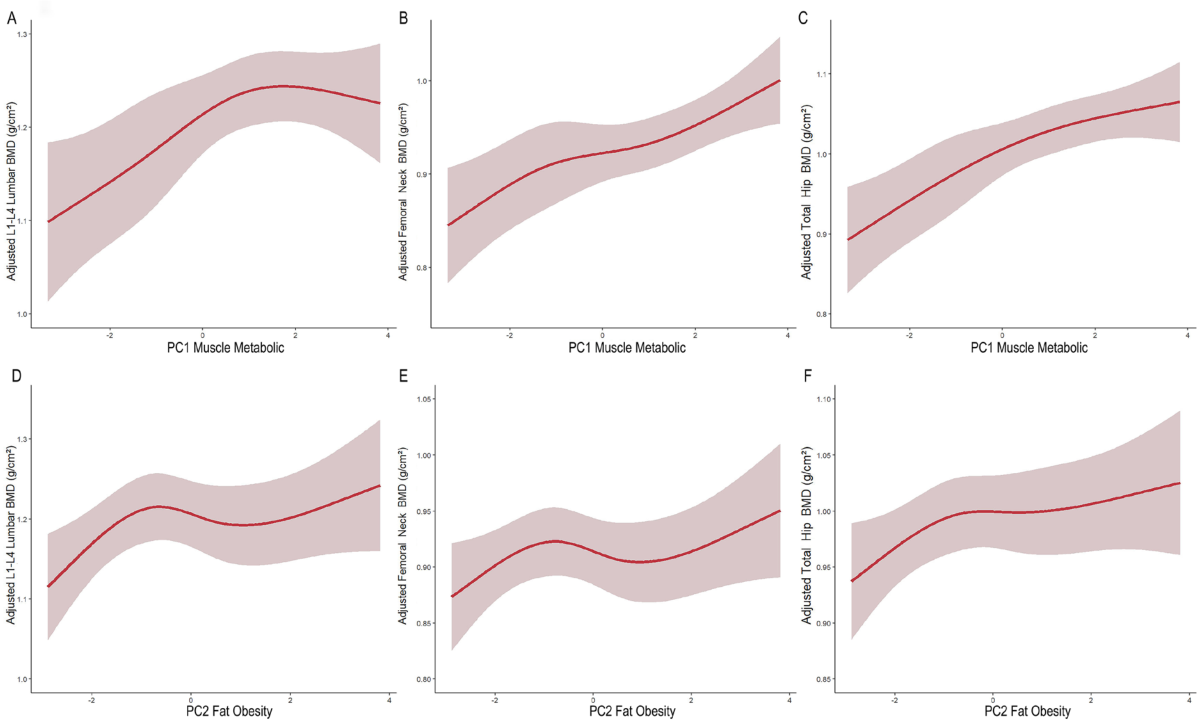

Supplementary Fig. S3 Nonlinear Associations of Body Composition PC1 and PC2 with BMD

RCS were used to model the potential non-linear relationships between PC1, PC2, and L1-L4 lumbar, femoral neck, and total hip BMD. The solid lines represent the predicted BMD values after adjustment for age, sex, smoking status, and diabetes duration, with the shaded areas indicating the 95% confidence intervals; The tests for non-linearity were non-significant for all models ((A) PC1-L1-L4 Lumbar: P =0.0647; (B) PC1-Femoral neck: P =0.5591; (C) PC1-Total hip: P = 0.2733; (D) PC2- L1-L4 Lumbar: P = 0.0551; (E) PC2-Femoral neck: P = 0.1275; (F) PC2-Total hip: P = 0.3523), providing strong evidence for linear associations and supporting the treatment of these components as linear variables in the multivariable linear regression models; Abbreviation: PC, principal component; BMD, bone mineral density; RCS, Restricted Cubic Spline.

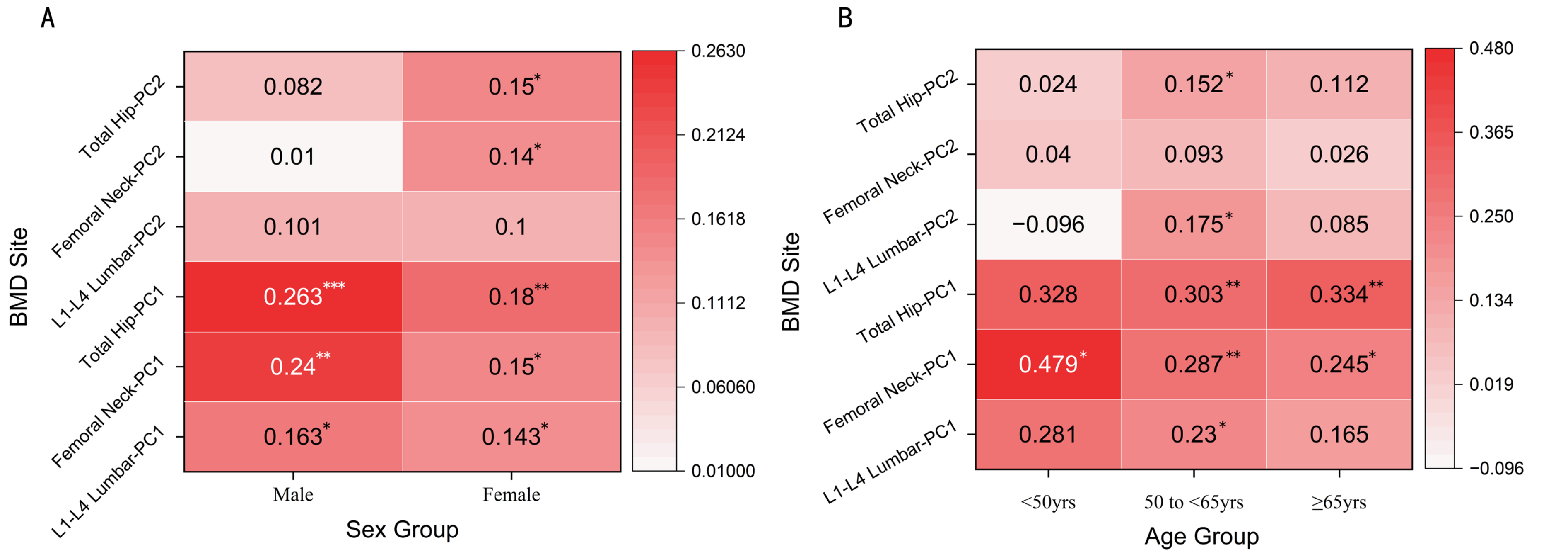

Supplementary Fig. S4 Sex-/ Age-Stratified Heatmap of Body Composition Effects on BMD

(A) Stratified analysis by sex group of the associations between body composition principal components and BMD at different sites; (B) Stratified analysis by age group (<50 years, 50 to <65 years, ≥65 years) of the associations between body composition principal components and BMD at different sites; Data in both heatmaps are presented as standardized β coefficients with significance levels denoted by asterisks (***P<0.001, ** P<0.01, * P <0.05); Abbreviation: PC, principal component; BMD, bone mineral density.

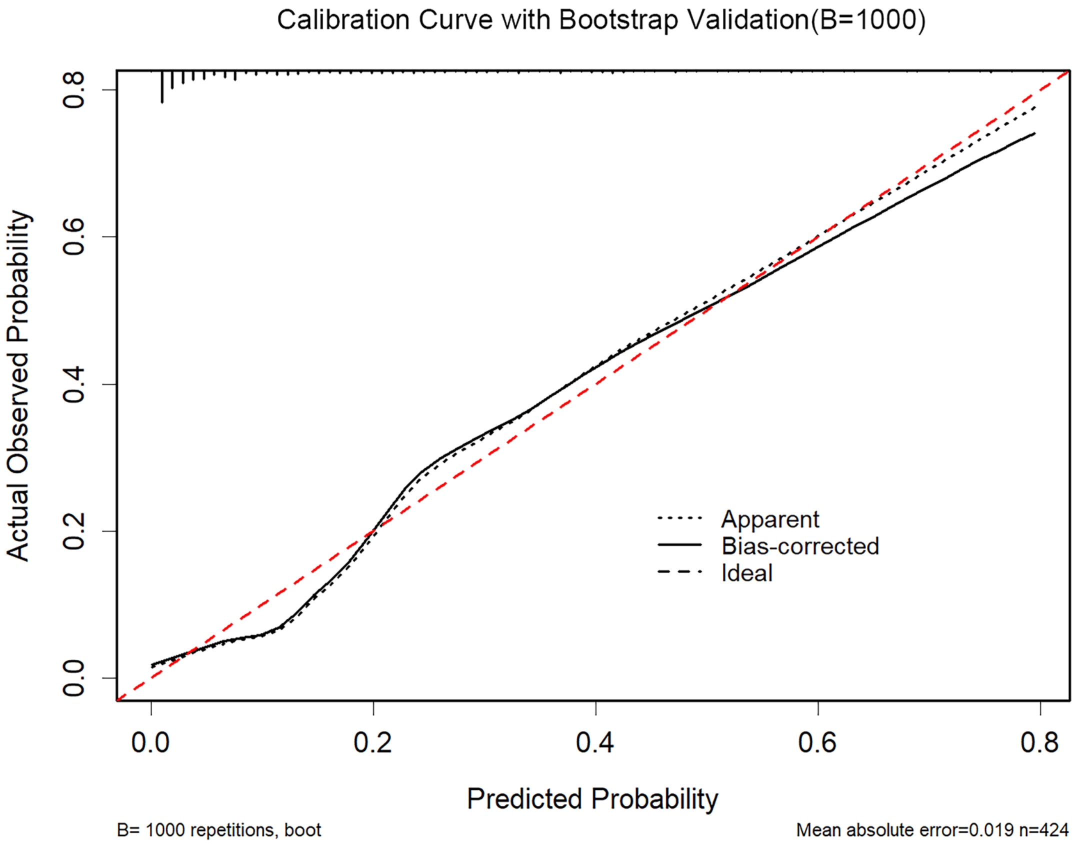

Supplementary Fig. S5 Calibration Curve of the Diagnostic Model

The calibration curve shows the relationship between the predicted probabilities and the observed frequencies. The dashed diagonal line represents perfect calibration. The solid line depicts the actual calibration of the model. The histogram illustrates the distribution of estimated probabilities. The mean absolute error is 0.019, indicating strong calibration performance.
